# Supplementary material for: Electrically empowered microcomb laser
Source: Nat Commun. 2024 May 17;15:4192. doi: 10.1038/s41467-024-48544-2 (PMC11101629; doi:10.1038/s41467-024-48544-2)
Supplement: Supplementary file 1 — Supplementary Information [file 41467_2024_48544_MOESM1_ESM.pdf]

Supplementary Information for
"Electrically enpowered micro-comb laser"

Jingwei Ling,[1,*] Zhengdong Gao,[1,*] Shixin Xue,[1] Qili Hu,[2] Mingxiao Li,[1] Kaibo Zhang,[2] Usman A. Javid,[2] Raymond Lopez-Rios,[2] Jeremy Staffa,[2] and Qiang Lin[1,2,†]

[1]*Department of Electrical and Computer Engineering, University of Rochester, Rochester, NY 14627, USA*
[2]*Institute of Optics, University of Rochester, Rochester, NY 14627, USA*

In this supplementary material, we provide detailed information on the comb laser design, characterization, data processing, and theoretical modeling.

I. LN DEVICE CHARACTERIZATION

Our LN PIC is based on a directly etched waveguide structure depicted in Fig. S.1**a**, with LN layer thickness of 600 nm. The CE comb laser and the FP comb laser have slightly different etching depths for dispersion management (300 nm for the CE comb laser, and 350 nm for the FP comb laser). Due to the difficulty of measuring the separate properties of each component within the CE comb laser cavity, we fabricated and tested the individual components such as racetrack resonators and Sagnac mirrors on the same wafer using identical fabrication parameters.

In this section, we will introduce the detailed device geometry and characterize the passive properties of the individual components, which include the optical Q of racetrack resonators, the reflection spectrum of Sagnac mirror, the dispersion of racetrack resonator and that of the main laser cavities. All the measurements are done in the telecom band.

A. Racetrack resonator

Here, we show the measurement results of the racetrack resonator for two different CE comb laser devices α and β. The device α with a racetrack waveguide wide of 1.6 μm is used for the results shown in Fig. 2, Fig. 4, and Fig. 5 in the main text. Another device β with a waveguide wide of 1.5 μm is shown as a comparison which will be further investigated in the following section.

We individually tested the transmission of the racetrack resonators with an external telecom tunable laser. The first parameter to characterize is the optical Q of the racetracks. Measuring the transmission trace of a critical coupled racetrack resonator with a width of 1.6 μm, we obtained an intrinsic Q of 1.6 million as shown in Fig. S.1**b**. Optical Q at a similar level is obtained for the 1.5-μm racetrack β. We then characterized the dispersion of the racetrack resonators by measuring the cavity resonance frequency ω_μ within a wavelength range from 1500 nm to 1600 nm, around the reference frequency ω_0 that corresponds to the wavelength at 1550 nm [1, 2]. The cavity resonance frequency follows the relationship, $\omega_\mu = \omega_0 + \mu D_1 + \frac{1}{2}\mu^2 D_2 + \frac{1}{6}\mu^3 D_3 + \cdots$, where $D_1/(2\pi)$ is the FSR around ω_0 and D_2 is related to the group velocity dispersion (GVD) parameter β_2 as $D_2 = -\frac{c}{n}D_1^2\beta_2$. Figure S.1**c** and **d** shows the experimentally recorded $\Delta\omega_\mu = \omega_\mu - \omega_0$ for Device α and β respectively, from which we obtained $\beta_2 = -0.02$ ps^2/m for the 1.6-μm racetrack α and $\beta_2 = -0.01$ ps^2/m for the 1.5-μm racetrack β.

B. Sagnac mirror

The schematic of the adiabatically coupled Sagnac mirror is depicted in Fig. S.1**e**. We adopt different Sagnac mirror designs for different comb laser devices based on the dispersion and reflectance requirements. A typical Sagnac mirror used for the CE comb laser in the main text has a long adiabatic coupling section[3] (L = 600 μm), with 300-nm etching depth, 0.8-μm width W and 0.3-μm gap between the waveguide. An adiabatic smooth transition to the waveguide of $\mathrm{W} \pm \mathrm{dW} = 0.9/0.7\mu$m is designed to be the coupling region. This adiabatic transition at the coupling region enables a broadband 50%:50% power splitting for the coupler and near 100% broadband reflection for the Sagnac loop mirror. To test the device, we butt-coupled light into a stand-alone Sagnac mirror and measured its reflection spectrum which is shown in Fig. S.1**f**. The recorded reflection spectrum shows strong oscillation which simply results from the Fabry-Perot-type resonance formed between the waveguide input facet and the Sagnac mirror. The consistently high and uniform extinction ratio serves as a compelling evidence of the Sagnac mirror's broadband high reflection. The gradual wavelength dependence of the spectrum amplitude is due to factors related to testing, such as wavelength-dependence of the input optical power, slightly dispersive reflection, and the limited bandwidth of optical circulator.

C. Laser cavity

Dispersion of the comb laser cavity matters for comb generation, especially for the FP comb laser. The average dispersion of Sagnac mirror together with bus waveguides and RSOA is designed to be near zero. The near-zero dispersion facilitates comb generation through both EO modulation and FWM. This can also be characterized by measuring the FSR of resonances from the reflected oscillating spectrum. The fitting curve of $\Delta\omega_\mu$ is shown in Fig. S.1**g**, for the FP comb laser

* These authors contributed equally.
† qiang.lin@rochester.edu

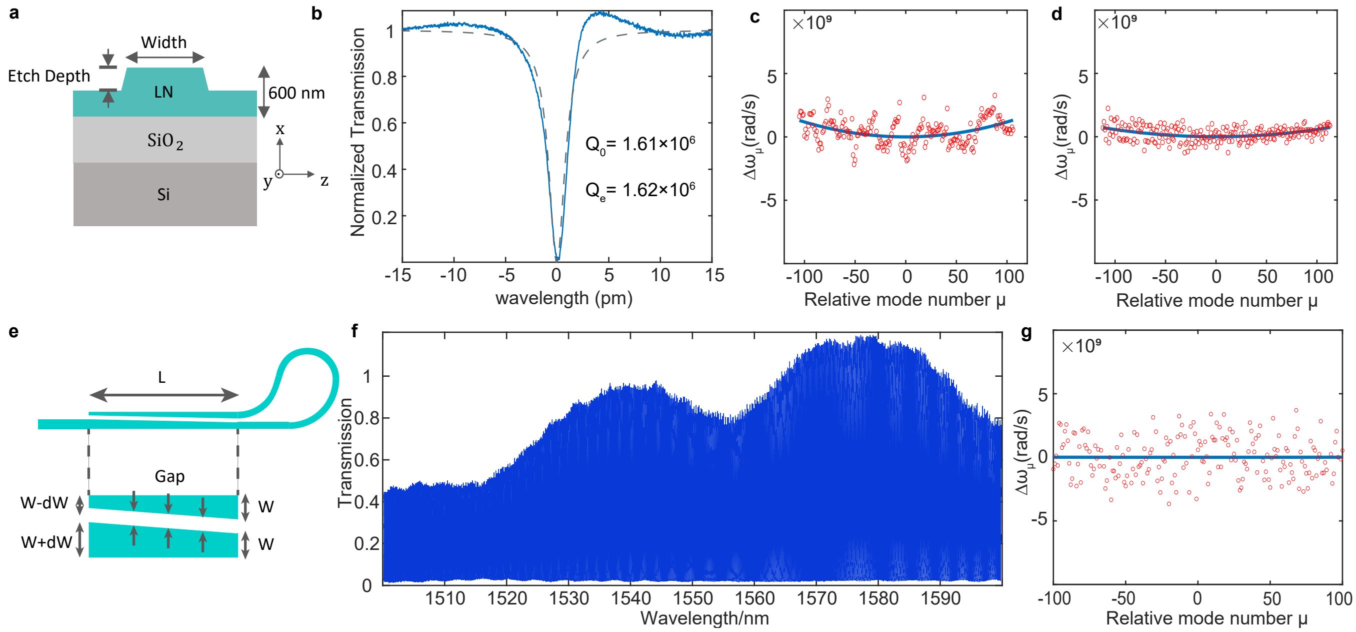

FIG. S.1. Individual components in the comb lasers and their passive performance. **a**, Schematic for the cross-section of the LN waveguide. **b**, Transmission curve of racetrack resonator with a width of 1.6 μm, critically coupled with a single bus waveguide. The dashed grey fitting curve corresponds to an intrinsic Q of 1.6 million. **c** and **d**, Mode dispersion curve versus the relative mode number, μ, for TE_{00} mode of resonator α and β. The red circles show the $\Delta\omega_\mu$ of each mode and the blue curves show the parabolic fittings. The GVD β_2 are fitted to be -0.02 ps^2/m for 1.6-μm-wide racetrack α in **c**, and -0.01 ps^2/m for 1.5-μm-wide racetrack β in **d**. **e**, Schematic of the adiabatic Sagnac loop mirror. **f**, Reflection spectrum from loop mirror measurement. **g**, Mode dispersion curve for the TE_{00} mode of total FP laser cavity, showing a β_2 of -0.003 ps^2/m.

device used in the main text. Due to the larger linewidth of the FP cavity resonance, slightly larger fluctuation is observed on the spectrum. Nevertheless, the averaged dispersion parameter β_2 is characterized to be -0.003 ps^2/m, a value that is close to zero.

On the other hand, CE comb lasers contain two cavities, the racetrack resonator, and the main laser cavity, the former of which dominates the comb generation process, with dispersion characterized in the previous section. We also minimize the average dispersion of the main laser cavity by designing the Sagnac mirror, and bus waveguides.

II. LASER PERFORMANCE ANALYSIS

In this section, we provide additional information regarding the performance of the comb lasers. We will show first alternate comb states, followed by two different CE comb lasers' behavior under varying RF driving signals. Then we will show the comb laser device working at a passive mode-locking state, with no EO driving. Furthermore, this section will incorporate details not addressed in the main text, including the linewidth measurements of comb and the approach adopted for comb tuning and data processing.

A. Performance of different comb lasers

The typical comb lasing states of device α are demonstrated in Fig.2 of the main text (marked as α). We also designed another comb laser (marked as β) with different dispersion and tested its comb lasing performance. The design differences between these two devices have been elaborated in the previous section.

We show here the performance of the device β with an FSR of 43.90 GHz. The comb outputs from the two different devices differ in the spectrum: Laser α features a comb output with two sidelobes; while Laser β has a single envelope with a sech^2 shape. As can be seen from Fig. S.2**a**, the sech^2 comb has a bandwidth of 7 nm. The spectrum of the comb-line beating note given in Fig. S.2**b** shows a clear single beating tone at 43.9 GHz with no extra tone or DC noise, showcasing the clear phase locking. These findings align well with the theoretical prediction, which will be discussed in Fig. S.9 of the following section. Noticeably, half-harmonic RF line doesn't appear in this case which indicates that it may relate to the unknown dynamics in Laser α.

B. Reconfiguration of comb lasers

To further investigate the dynamics and performance, and to show the reconfigurability of the comb laser, we tested the performance of CE comb laser α and β under different RF

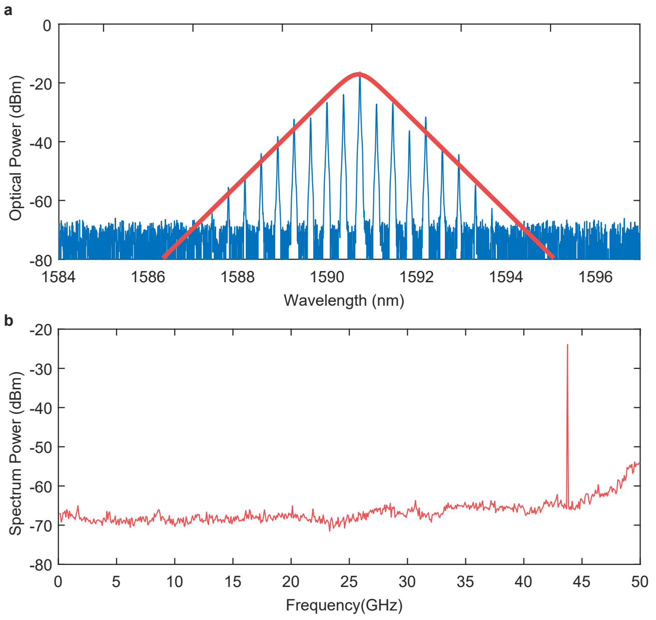

FIG. S.2. Optical comb generated from Laser β. **a**, Optical spectrum of the generated comb, The blue comb spectrum is fitted with red sech^2 curve. **b**, Electrical spectrum of the beat note detected from the comb laser output, with single tone at one FSR. DC background or half-harmonic tone is not observed in the spectrum.

driving signals. The optical bandwidths and mode-lock states can be adjusted with different electrical inputs. With the CE comb laser device α shown in the main text, we fixed the current at 270 mA and measured the optical spectra of the comb at different driving RF powers and RF frequencies. As evident from Fig. S.3**b-c**, increasing the power of the RF driving signal leads to an expansion of the comb spectrum and a corresponding rise in output power. Of particular interest is that when the driving current is held constant, the RF driving and comb bandwidth influence not only the comb power of the laser but also the output of RSOA itself. Such an intriguing enhancement effect accounts partially for the high wall-plug efficiency we mentioned in the main text.

The repetition rate and comb FSR can be slightly tuned by varying the driving frequency. To quantitatively characterize this effect, we examined Laser α output spectrum versus the driving frequency deviation from the racetrack resonator's FSR. An increase in the detuning of the driving RF frequency from the resonator FSR leads to a reduction in output comb bandwidth, as evident in Fig. S.3**e**. Specifically for Laser α, greatly increased detuning alters the comb shape from two sidelobes to a single lobe.

The reconfigurability of the comb laser is further verified with the other CE comb laser β. Although the two devices differ in design, and thus different dispersion and comb shapes, they exhibit consistent trends in response to the changes in the RF driving power and frequency. The consistencies are proved by Fig. S.3**f-h** and Fig. S.3**i-j**, for RF power and frequency, respectively.

Sharing the similar comb evolution behavior with driving RF signals change, the two laser devices are still drastically different in the comb spectra. The varied dispersion and cavity roundtrips may be the primary contributors to the different comb generation in Fig. S.3. In Laser β, we notice the comb output spectra are more localized with narrower spectra bandwidths, while the comb spectral envelopes of Laser α tend to shift around with different RF drivings. One possible explanation is that better group delay matching is achieved between the racetrack resonator and main laser cavity for device α, which allows for the formation of a broader range of comb lines at different wavelengths. However, the details of the mechanism behind require further study.

C. Passive mode locking

The comb laser is also able to operate without the EO driving or initiation in which the Kerr-based FWM serves as the mode-locking mechanism. With another CE comb laser (marked as γ) which has a racetrack resonator with lower coupling loss and higher loaded Q, we observed such passive mode locking as shown in Fig. S.4. Despite a narrower bandwidth than that shown in Fig. S.2, the comb spectrum also features a sech^2 spectral shape as shown by the fitted red curve. The comb state also exhibits a clean electrical comb-line beating tone and time-domain pulses measured by autocorrelation (not shown), as an indication of phase-locking. The spectral bandwidth of the passive mode-locked comb is mainly limited by the RSOA output power and the roundtrip mismatch issue we described in the discussion section of the main text.

D. Spectrum of the comb-line beat note around the DC region

In the main text, we have shown the comprehensive spectrum of the comb-line beating node over a broad bandwidth for the comb produced by the CE comb laser α. Here, we present a detailed view of this spectrum, for the frequency region from DC to 1 GHz. For comparison, we also include in Fig. S.5 both spectra of the background noise originating from the photodetector and of the single-mode lasing state. Remarkably, these three curves exhibit a complete overlap. The low noise around the DC region serves as a compelling evidence of effective phase locking that exists among all the comb lines.

E. Comb linewidth measurement

The linewidth measurement is performed using the sub-coherent length self-heterodyne method [4–6]. The setup is shown in Fig. S.6, in which a balanced PD is used for suppressing the detectors' common noise. We use an AOM to frequency shift the whole comb by 55 MHz and heterodyne the comb with itself after sub-coherent delay. Since the delay length is less than the coherent length, the heterodyned optical signals are not entirely independent. The phase noise and

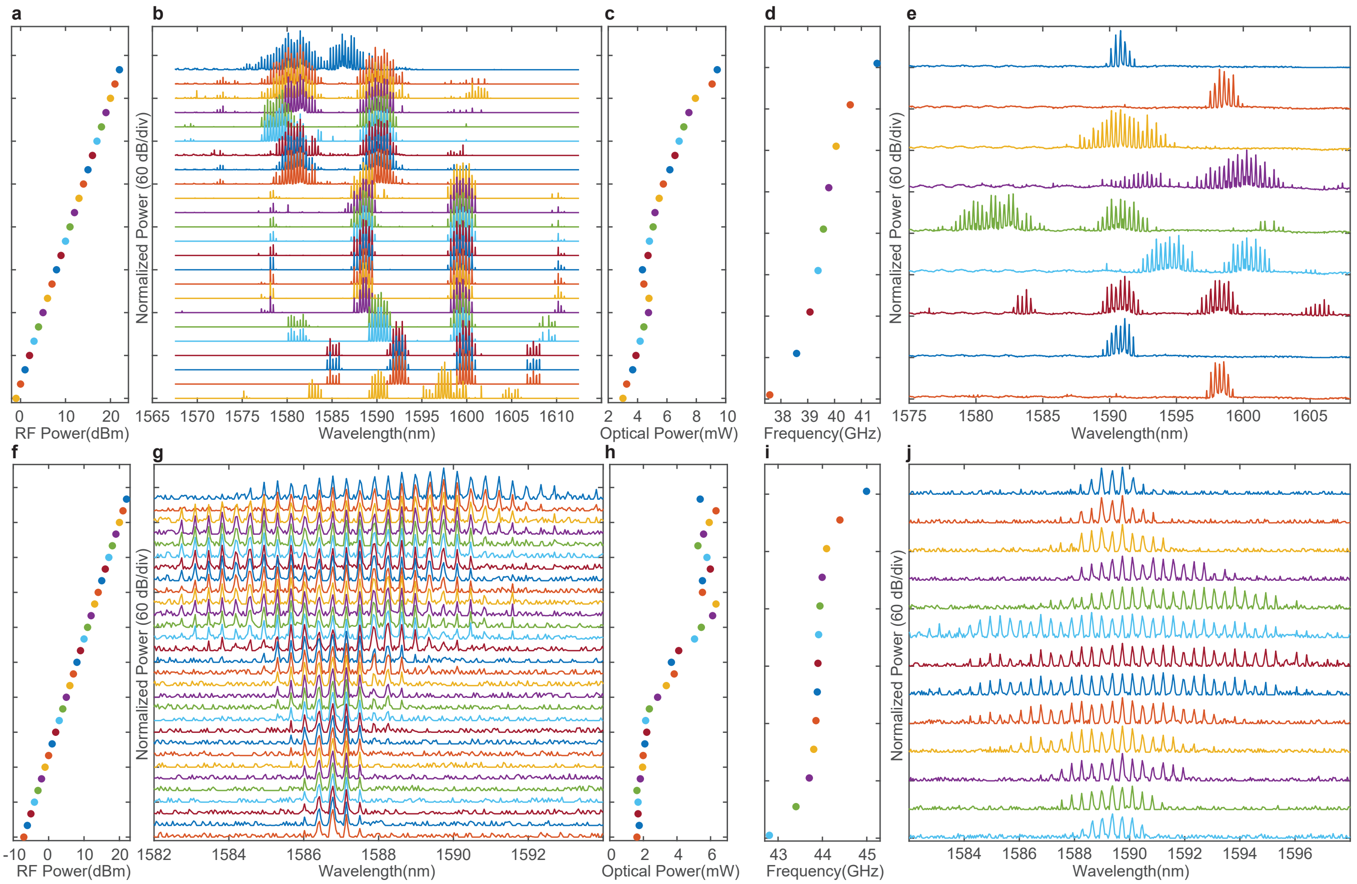

FIG. S.3. Reconfiguration of CE comb lasers. Comb spectra at different RF powers and frequencies are demonstrated, for device α (FSR=39.58 GHz) in **a**-**e** and for device β (FSR= 43.90 GHz) in **f**-**j**. **a**-**c**, Performances of Laser α with 39.58 GHz RF driving but different RF powers: **a**, Input RF driving power; **b**, Normalized comb output spectra; **c**, Output optical power, growing with increased RF input. **d**-**e**, Performances of Laser α with 20 dBm RF driving at different frequencies: **d**, Input RF driving frequency; **e**, Normalized comb output spectra at different driving frequencies, bandwidth maximizes at one FSR. **f**-**h**, Performances of Laser β with 43.90 GHz RF driving but different RF powers: **f**, Input RF driving power; **g**, Normalized comb output spectra; **h**, Output optical power with increased RF input, growing with increased RF driving and saturating at 6mW. **i**-**j**, Performances of Laser β with 20 dBm RF driving at different frequencies: **i**, Input RF driving frequency; **j**, Normalized comb output spectra at different driving frequencies, bandwidth maximizes at one FSR.

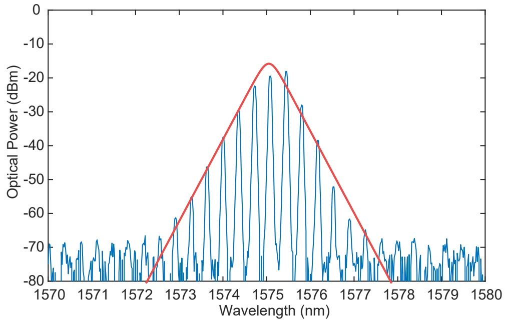

FIG. S.4. Optical comb generated from passive mode locking in CE comb laser, with no EO modulation applied. The blue comb spectrum is fitted with red sech^2 curve.

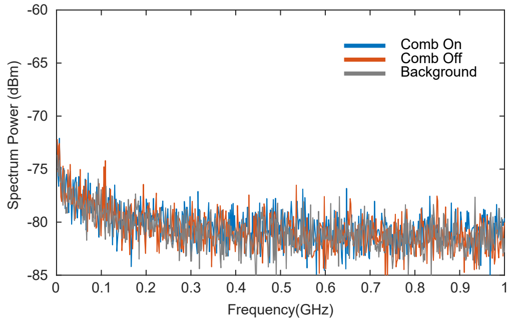

FIG. S.5. Zoom-in Electrical spectrum of the CE comb laser output beating signal, ranging from DC to 1 GHz.

linewidth are recovered by the following relation:

$$S_\phi(f) = S_{\Delta\phi}(f) \cdot \frac{1}{4\sin^2(\pi f \tau)} = \frac{\delta\nu}{\pi f^2}, \qquad \text{(S.1)}$$

where S_ϕ is the phase noise of the comb laser, $S_{\Delta\phi}$ is the phase noise measured from the sub-coherent heterodyne measurement, and $\delta\nu$ is the Lorentzian linewidth. We performed the

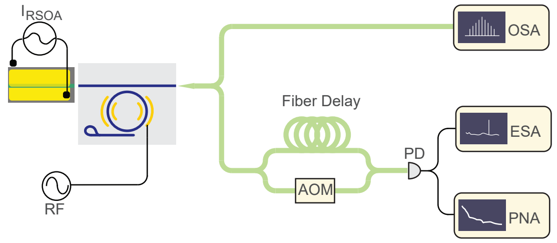

FIG. S.6. Experimental setup for measuring the linewidth of the comb. Sub-coherent length self-heterodyne method is used. The frequency is shifted by 55 MHz with an AOM , and single-mode fiber delays with 30 meter are used for heterodyne. AOM: acoustic optical modulator; OSA: optical spectrum analyzer; PD: photo-detector; ESA: electrical spectrum analyzer; PNA: phase noise analyzer.

measurement with different delays from single-mode fibers with different lengths of 17 m, 30 m, and 1 km, and obtained the same intrinsic linewidth. The results shown in the main text are measured with a 30-m delay fiber.

Noticeably, the measured linewidth represents the overall linewidth contributed from the entire comb, which indicates the upper limit of the intrinsic linewidth, as the entire comb is self-heterodyned without any filtering. We employ this method to more effectively showcase the overall coherence of the comb and to further substantiate the phase-locking among all comb lines.

F. Fast frequency tuning of comb

In the experiment of high-speed comb frequency tuning described in the main text, the comb frequency modulation was measured by recording the temporal waveform of a beating signal between the comb and an external single-frequency CW laser near 1582 nm (see Fig. S.7). The external laser is tunable to control the beating node frequency, which was chosen to be 15 GHz as the carrier frequency in the experiment. When the triangular driving signal is turned off, the beating signal appears as a sinusoidal temporal waveform with a constant frequency of 15 GHz, similar to the trace in Fig. 2**h** of the main text. The triangular driving signal introduces chirping to the beating signal. We recorded the real-time beating signal and retrieved the time-frequency spectrum through the short-time Fourier transform (STFT). The results are shown in Fig. 4**b-d** of the main text.

The time-frequency spectra exhibit certain distortions on its time-dependent waveforms, which primarily arise from two factors. First, the waveform of the driving electric signal is distorted by some extent from an ideal triangular waveform, due to the limited bandwidth of the RF amplifier used to boost its amplitude, as evident on the dashed curves in Fig. 4**b-d** of the main text. This part of waveform distortion is transferred directly to the comb frequency tuning waveform. Second, the time window used in the STFT introduces rounding and distortion particularly around the transition regions between frequency up-chirping and down-chirping. On the other hand, the time-frequency spectra also exhibit certain blurring on the

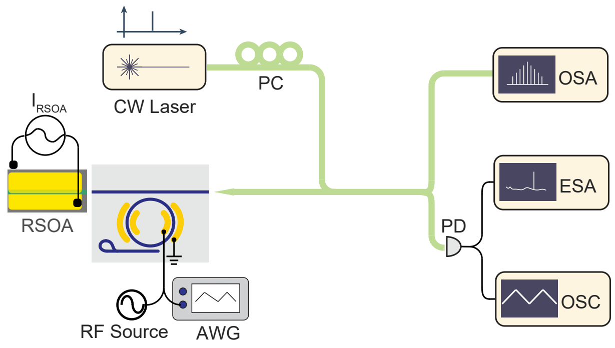

FIG. S.7. Experimental setup for the comb tuning experiment. An arbitrary waveform generator (AWG) is used for modulating the comb laser. The comb output is beaten with an external CW laser at 1582 nm, and the real-time beating signal from PD is recorded by the OSC. Comb tuning is recovered from the post-processing of the beating signal. PC: polarization controller; OSC: real-time oscilloscope

waveform particualry at a high modulation speed, which is simply due to the limited time window used for STFT.

To determine the waveform deviation between the driving electrical signal and the comb frequency tuning, we find the spectral peak of a time-frequency spectrum at every time point, which forms the time-dependent frequency tuning curve of the comb. We compare it with the recorded time-dependent waveform of the driving electrical signal. For a fair comparison, rounding effect due to the limited time window is taken into account for the waveform of the driving electric signal. By comparing the two curves, we obtained the waveform deviation curve shown in the lower panels of Fig. 4**b-d** of the main text.

III. THEORETICAL MODELING

In the main text, we provide a comprehensive description of the design and experimental implementation. Here, we employ a simplified model to elucidate the underlying physical mechanisms of the comb laser operation. Fig. S.8 provides the simplified schematics of the comb laser systems, including a conceptual overview of the key components for both the cavity-enhanced (CE) and Fabry-Perot (FP) comb lasers. In this section, we will delve into the system dynamics and aim to substantiate the experimental findings through mathematical analysis and numerical simulations.

A. Dynamics of comb laser

The comb lasing dynamics involves EO modulation and FWM process inside the laser cavity. Here we analyze the cavity-enhanced comb laser as an example. The FP comb laser can be analyzed using a similar approach by removing corresponding terms. The physical configuration of the cavity-enhanced laser is shown in Fig. S.8**a**, highlighting its

three primary components: the RSOA, the resonator, and the Sagnac mirror. The electrodes at the resonator adopt a Ground-Signal-Signal-Ground (GSSG) configuration and are modulating at one FSR of the racetrack, thereby introducing the coupling between adjacent resonant modes.

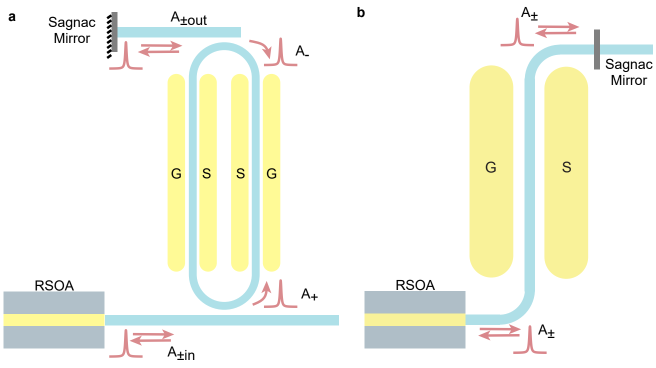

FIG. S.8. Device schematics. **a,** Cavity-enhanced CE comb laser device consists of RSOA, EO modulated resonator, and broad-band Sagnac mirror. The optical field of the resonator and the lower/upper bus waveguide are represented by A, A_{in} and A_{out} respectively. **b,** FP comb laser device, the laser cavity only includes RSOA, phase-shifting waveguide section, and Sagnac mirror. The optical field inside the laser is represented by A.

To quantitatively analyze the dynamics, we use a model similar to the nonlinear Shrödinger equation (NLS) and the Lugiato–Lefever equation (LLE) and try to study the dynamics majorly in the time domain for the intracavity waveform of the racetrack resonator. We first consider the combined effect of Kerr and laser gain, and the EO modulation will be included next as an extra term for the modified LLE. The optical field at the resonator and the two bus waveguides can be written in $A_{\pm}$, $A_{\pm in}$ and $A_{\pm out}$ as shown in Fig. S.8**a**, where "+" corresponds to the counter-clockwise (CCW) wave and "-" represents the clockwise (CW) wave. Considering that the Sagnac mirror has high flat reflectance, the CW wave and CCW wave have no nonlinear interaction inside the cavity, we can assume that the CW wave and CCW wave are identical except for fixed-length beam propagation. Also, since the power inside the racetrack resonator is significantly higher than the main laser cavity, we simplify the model by neglecting the nonlinearity of the main laser cavity. Besides, we combine the nonlinear process of CCW and CW resonant modes and only study them in one direction. Symmetric coupling conditions ($\theta = \theta_1 = \theta_2$) for the different bus waveguides to resonator coupling are also assumed in this analysis. As the optical field of the CW direction remains identical to that of the CCW direction plus beam propagation, we only study A_+ in the CCW direction and rewrite it as A, with the following relations:

$$t_R\frac{\partial A(t,\tau)}{\partial t} =\Big[-\alpha - i\delta_0 + iL\sum_{k\geq 2}\frac{\beta_k}{k!}(i\frac{\partial}{\partial\tau})^k + i\gamma L|A|^2\Big]A + \sqrt{\theta}A_{in}, \tag{S.2}$$

$$A_{in}(t,\tau) =G_R\cdot(1+iL_l\sum_{k\geq 2}\frac{\beta_{kl}}{k!}(i\frac{\partial}{\partial\tau})^k)\cdot A(t,\tau-t_{Rl}), \tag{S.3}$$

$$G_R =e^{-\alpha_l}\cdot\theta^2\cdot R(\omega)\cdot\frac{g_m(\omega)}{1+|\bar{A}|^2/|A_{sat}|^2}. \tag{S.4}$$

The physical process can be broken down into three equations. The Eq. S.2 is the LLE equation of the racetrack resonator[7]. Here, A is the optical field inside the racetrack such that $|A|^2$ is normalized to optical power, and A_{in} is the optical field inside the bus waveguide as the input for the resonator. The detuning δ_0 is zeroed when the racetrack resonator central mode matches with the main laser cavity. The optical field change in the bus waveguide per main laser cavity roundtrip is discussed in Eq. S.3 and Eq. S.4. For the bus waveguide of main laser cavity, the light coupled into and out of the racetrack twice per roundtrip, reflected once by the Sagnac mirror and amplified once by the RSOA, all of these beam propagation processes are written together as roundtrip gain G_R in the Eq. S.3. Meanwhile, the dispersion of beam propagation of the bus waveguide is also included as the term $iL_l\sum_{k\geq 2}\frac{\beta_{kl}}{k!}(i\frac{\partial}{\partial\tau})^k$. The roundtrip gain G_R, on the other hand, is discussed by Eq. S.4, and is related to loss, reflectance, and saturated RSOA gain.

In these equations, t_R and t_{Rl} is the roundtrip time of the racetrack resonator and the laser main cavity, L and L_l is the normlized cavity length of the racetrack and the laser main cavity. $\alpha = \alpha_0/2+\theta_1/2+\theta_2/2$ is the total loss (where α_0 is the intrinsic absorption coefficient and θ is the coupling coefficient between the resonator and the two bus waveguide), α_l is the loss of the laser main cavity. β_k and β_{kl} is the averaged k^{th} order dispersion coefficient of the resonator and laser main cavity, $\gamma = \frac{n_2\omega_0}{cA_{\text{eff}}}$ is the nonlinear coefficient from Kerr effect. G_R denotes the roundtrip gain of the laser, where R is the reflectance of the Sagnac mirror and RSOA, g_m and A_{sat} here are the small signal gain and the saturated optical field of RSOA. The wavelength dependency of gain $g_m(\omega)$ and reflection $R(\omega)$ are addressed as function, but the time response of RSOA is considered to be instantaneous in this simplified model.

The above equations cover the dynamics for passive mode locking and here we include EO modulation as a fast time-dependent phase modulation. When EO modulation occurs at the k-th order of the racetrack resonator's FSR, the modified LLE equation Eq. S.2 for comb laser can be re-write as the following:

$$t_R\frac{\partial A(t,\tau)}{\partial t} =\Big[-\alpha - i\delta_0 + iL\sum_{k\geq 2}\frac{\beta_k}{k!}(i\frac{\partial}{\partial\tau})^k + i\gamma L|A|^2 + i\eta_{EO}E_0cos(\frac{2k\pi\tau}{t_R})\Big]A + \sqrt{\theta}A_{in}, \tag{S.5}$$

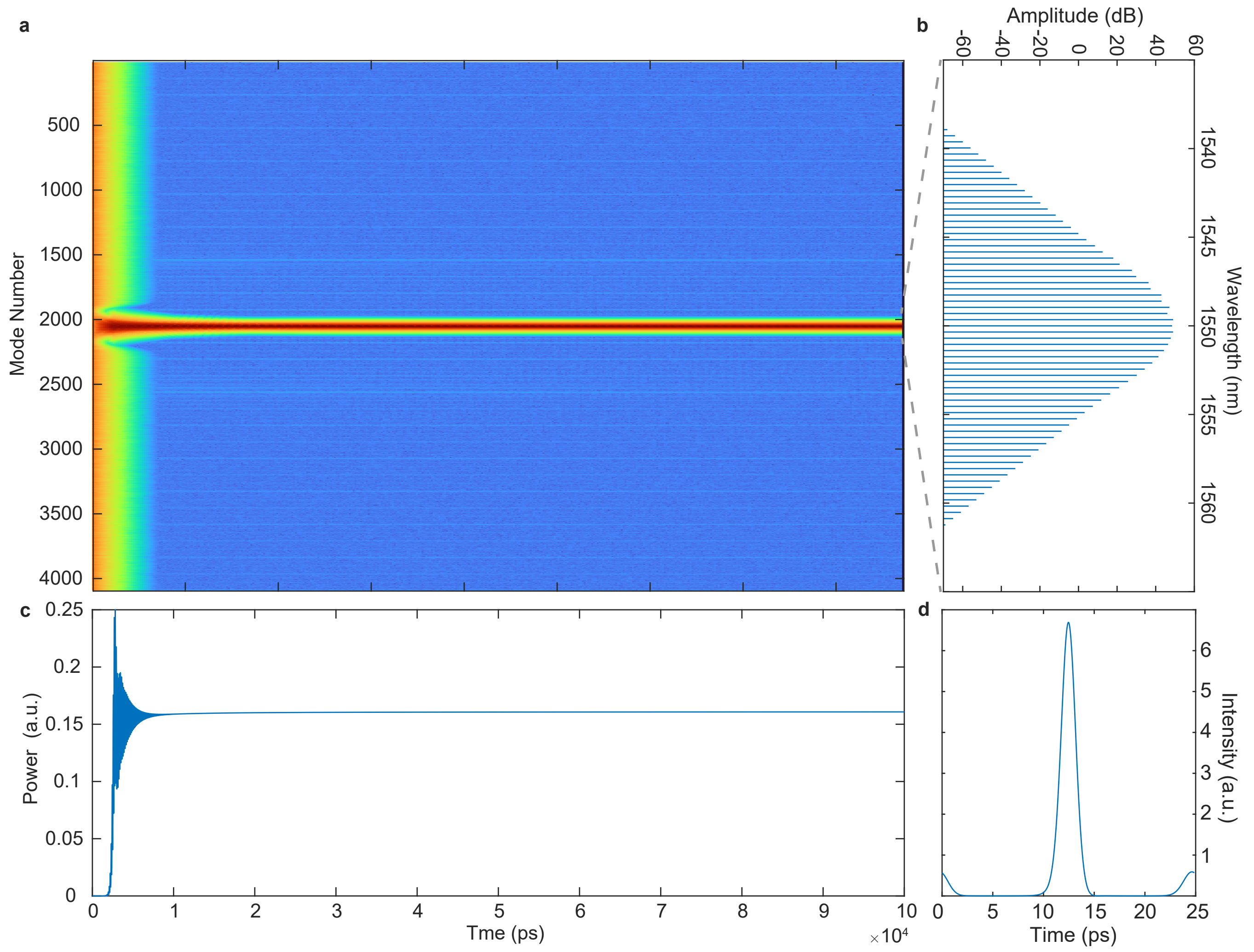

FIG. S.9. Simulation result for the turning-on process of a CE comb laser. The gain is turned on at time zero, with a constant EO driving frequency of one FSR. **a,** Time-dependent spectrum evolution of the comb self-starting process. **b,** Final spectrum of the lasing soliton comb. **c,** Time-dependent intra-cavity power evolution of the comb self-starting process. **d,** Final time domain pulse shape of the lasing soliton.

where η_{EO} is the EO coefficient and E_0 is the electrical field strength, the combination of which forms the non-dimensional phase change. A simple model of cosine phase oscillation is used here to represent the EO modulation. After introducing the EO effect, the Eq. S.5, Eq. S.4 and Eq. S.3 form a complete set for the theoretical description of the CE comb laser.

B. Simulation of comb laser self-starting

With the above theoretical model, the numerical simulation can be run using the split-step Fourier method. This simulation only considers key physical elements mentioned above, with simplification on other dynamics including thermal nonlinearity, photo-refraction, amplifier response and others. As can be seen in the Fig. S.9. The figure shows the activation process of the laser with a constant EO driving signal of one FSR applied on the electrodes. The comb laser used in this simulation has an intrinsic optical Q of 1.5 million, coupling Q of 3 million, β_2 of the resonator and β_{2l} of the main laser cavity are -0.02 ps^2/m and 0.05 ps^2/m, respectively. The main laser cavity optical path is exactly 3 times the racetrack resonator's.

As can be seen in Fig. S.9**a** and **c**, when the laser gain is turned on, a chaotic comb is started at first but quickly settled to a stable state, which ends at the soliton states shown by Fig. S.9**b** and **d**, with the noises greatly suppressed. From the power diagram, the laser power first climbs up and rings after turning on and quickly stabilizes to a constant value. The spectrum of the final stable single soliton state features a sech^2 shape with a bandwidth around 20 nm, as shown in Fig. S.9**b**. Such a shape is similar to the testing results of Laser β in the previous section. The corresponding time domain pulse shape in Fig. S.9**d** is also consistent with the autocorrelation trace obtained experimentally. It's particularly noteworthy that, unlike the Continuous Wave (CW) pumped frequency comb that features a central residual comb line, the lased soliton comb in this system exhibits no CW background. This characteristic is

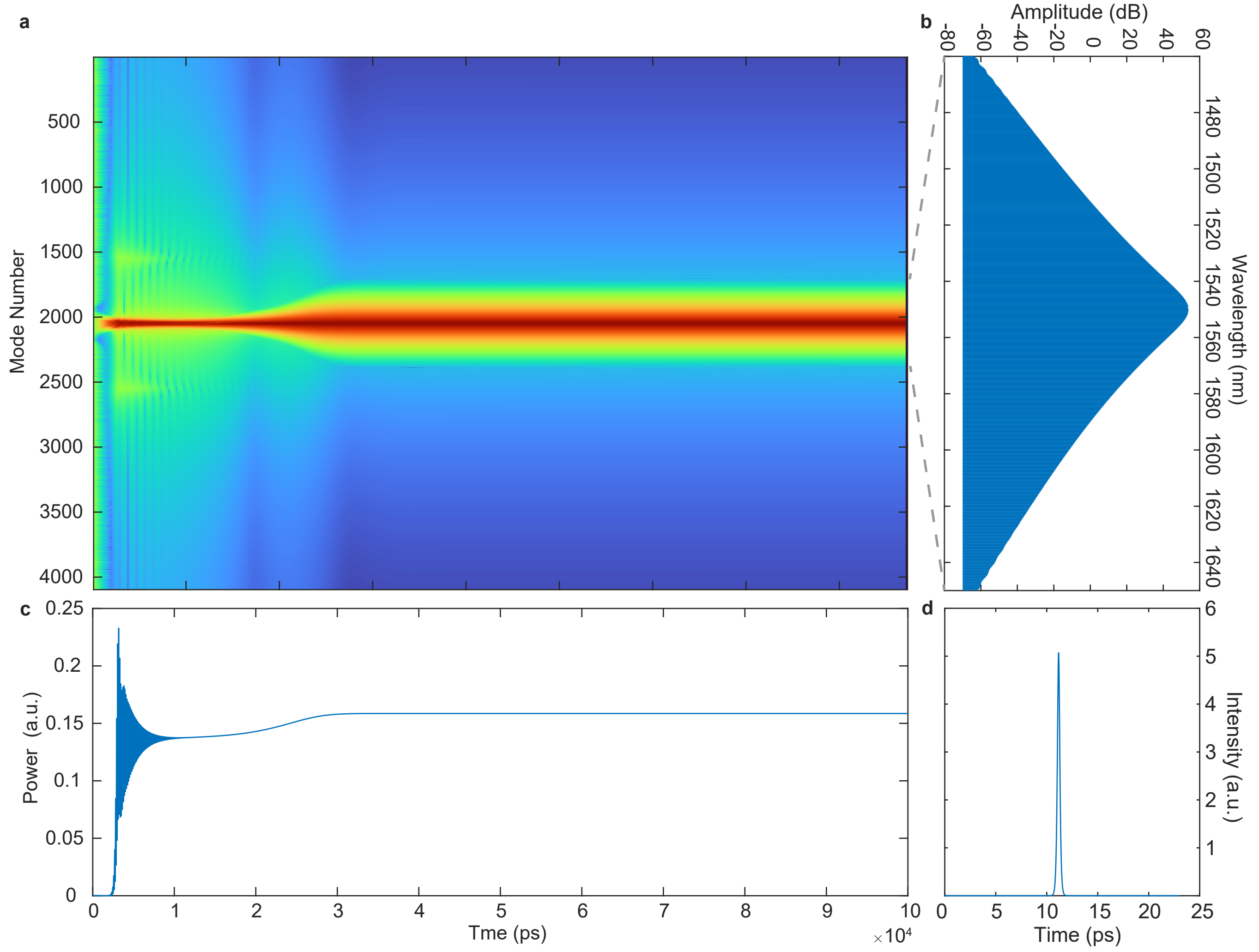

FIG. S.10. Simulation result for another example of turning on a CE comb laser, with stronger RSOA gain and significantly wider spectrum. **a,** Time-dependent spectrum evolution of the comb self-starting process. **b,** Final spectrum of the lasing soliton comb. **c,** Time-dependent intra-cavity power evolution of the comb self-starting process. **d,** Final time domain pulse shape of the lasing soliton.

evident in Fig. S.9**b** and **d** and aligns well with the experimental results presented in the main text. The soliton comb extracts all of its energy directly from the RSOA, thereby maximizing conversion efficiency.

Changing the RSOA gain or the maximum optical power output of RSOA, and fixing the EO driving, we can find another state of the CE comb laser output which features a significantly wider bandwidth. With increased optical power, the FWM expands the comb greatly, and the optical bandwidth of the comb increases to 150 nm in Fig. S.10**b**, leading to a shorter time domain pulse in Fig. S.10**d**. The time domain pulse shape also has a clean background and high extinction ratio, which is fundamentally different from normal Kerr cavity soliton. Changing the optical power, we are also able to control the bandwidth of the output spectrum in the simulation just like the example in Fig. 5 of the main text, and all the initiation processes stay robust. Additionally, this suggests that enhanced coupling, increased RSOA strength, and refined roundtrip time control could extend the optical bandwidth of the comb laser far beyond the current limit.

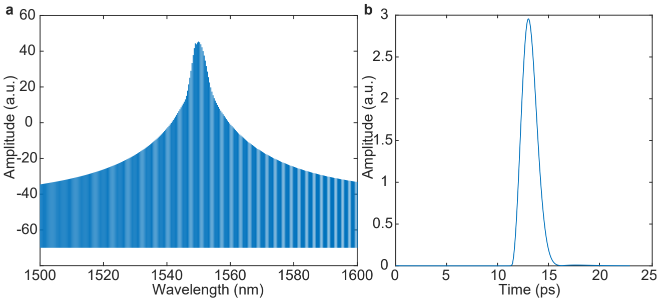

FIG. S.11. Simulation result for CE comb laser, with constant strong EO driving frequency at one FSR but low RSOA output power to suppress the FWM process. **a,** The final spectrum of the lasing EO comb. **b,** The final time domain pulse shape of the lasing EO comb.

C. EO comb lasing

The lasing comb also differs from the pure EO comb[8] drastically. EO modulation creates correlations between different laser modes to start multimode lasing which in turn serves as a means to initiate and lock the comb lasing, instead of directly distributing power from the center line. To better demonstrate this, we decrease the RSOA output power to a negligible level (no FWM) but increase the EO modulation power to form a lased EO comb as shown in Fig. S.11. Due to the low optical power, FWM barely participates in the process and the spectrum of the EO comb features a special shape shown in Fig. S.11**a**, with the corresponding pulse shape in Fig. S.11**b**. This EO comb laser resembles the traditional active mode-lock laser, where only EO modulation serves as the locking mechanism.

[1] Herr, T. *et al.* Mode spectrum and temporal soliton formation in optical microresonators. *Physical review letters* **113**, 123901 (2014).

[2] Yi, X., Yang, Q.-F., Yang, K. Y., Suh, M.-G. & Vahala, K. Soliton frequency comb at microwave rates in a high-Q silica microresonator. *Optica* **2**, 1078–1085 (2015).

[3] Ramadan, T. A. & Osgood, R. M. Adiabatic couplers: design rules and optimization. *Journal of lightwave technology* **16**, 277 (1998).

[4] Camatel, S. & Ferrero, V. Narrow linewidth cw laser phase noise characterization methods for coherent transmission system applications. *Journal of Lightwave Technology* **26**, 3048–3055 (2008).

[5] Yuan, Z. *et al.* Correlated self-heterodyne method for ultra-low-noise laser linewidth measurements. *Optics Express* **30**, 25147–25161 (2022).

[6] Tran, M. A., Huang, D. & Bowers, J. E. Tutorial on narrow linewidth tunable semiconductor lasers using si/iii-v heterogeneous integration. *APL photonics* **4** (2019).

[7] Coen, S. & Erkintalo, M. Universal scaling laws of kerr frequency combs. *Optics letters* **38**, 1790–1792 (2013).

[8] Zhang, M. *et al.* Broadband electro-optic frequency comb generation in a lithium niobate microring resonator. *Nature* **568**, 373–377 (2019).
